# China's Fight Against COVID-19: What We Have Done and What We Should Do Next?

**DOI:** 10.3389/fpubh.2022.548056

**Published:** 2022-06-21

**Authors:** Sixiang Cheng, Yuxin Zhao, Atipatsa Chiwanda Kaminga, Xinping Zhang, Huilan Xu

**Affiliations:** ^1^Department of Social Medicine and Health Management, Xiangya School of Public Health, Central South University, Changsha, China; ^2^College of Data Science and Information Engineering, Guizhou Minzu University, Guiyang, China; ^3^Department of Mathematics and Statistics, Mzuzu University, Mzuzu, Malawi; ^4^Department of Epidemiology and Health Statistics, Xiangya School of Public Health, Central South University, Changsha, China; ^5^School of Medicine and Health Management, Tongji Medical College of Huazhong University of Science and Technology, Wuhan, China

**Keywords:** China, COVID-19, intervention measures, change features, lessons

## Abstract

**Background:**

Chinese government conducted unprecedented massive public health prevention interventions at the national level, which have effectively contained the spread of Coronavirus Disease 2019 (COVID-19) infections. Specifically, the outbreak in Wuhan has been effectively controlled. Meanwhile, the Chinese efforts to contain the virus have been widely recognized. Even the World Health Organization has praised the efforts of the Chinese government and advised other countries to learn from China's experience in the fight against COVID-19. However, the measures that have been conducted by China to effectively prevent the spread of COVID 19 in the country have not been rigorously analyzed. Therefore, this study aimed to explore the characteristics of China's control and prevention strategies, and identify the elements that changed the epidemiological curve of rapidly rising new confirmed cases of COVID-19.

**Methods:**

Public health intervention measures and their effects on the spread of COVID-19 in terms of daily newly confirmed and cumulative cases were collected between January 20, 2020, and March 5. Notices of the Joint Prevention and Control Mechanism for COVID-19 of the State Council on Implementing Measures in Hubei were collected. Information obtained by relevant important documents and announcements was collected from the official website of the Chinese government. Additionally, from other media platforms, news, articles, and reviews were used to explain the intervention measure. Thus, using these data, we performed a retrospective description of the intervention strategies at three stages.

**Results:**

The Chinese government adopted non-pharmacological interventions measures (NPIs) timely and efficiently. On February 20, the declining epidemic trend in China indicated that the three strictest disease prevention and control strategies issued by the Hubei Government had contributed to a smooth decline in the spread of the epidemic.

**Conclusions:**

The NPIs taken by China play a decisive role to control the spread of novel coronavirus outbreaks. Further research and action are needed to ensure a sufficiently sensitive surveillance system and strong response mechanism, including the establishment of a highly accessible laboratory network, maintenance of awareness of both primary healthcare providers and the public, and regular training and exercise of local Centers for Disease Control and Prevention and general practitioners in the community-level.

## Introduction

An unprecedented outbreak of pneumonia of unknown etiology in Wuhan, Hubei Province, China, emerged in December 2019 (1, 2). However, this was identified as a symptom of COVID-19 disease, 2019-nCoV, caused by a novel coronavirus (3). The spread of COVID-19 turned into an epidemic when it quickly affected 31 provinces and regions of China. Later the spread of COVID-19 became a pandemic, when it was announced on March 5 at 23:00 CET that a total of 98,067 new cases were confirmed globally, with 3,281 deaths (4). Specifically, a total of about 17,637 cases were confirmed in 78 countries outside China. In this regard, the COVID-19 outbreak surpassed the severity of the severe acute respiratory syndrome coronavirus 2 (SARS-CoV-2) outbreak in 2003 (5–7).

In China, the COVID-19 outbreak coincided with China's Spring Festival travel peak. Therefore, to curb the spread of the infectious disease, the Chinese government launched unprecedented public health intervention measures such as shutting down Wuhan transportation, extending the legal holiday, mass isolation of individuals from cases, strict enforcement of quarantine of all individuals who had contact with the suspected cases, cancellation of all public gatherings, and educating the public on how to prevent the spread of the disease by practicing hygiene (8). In addition, a nationwide health monitoring of people who had visited the Hubei province or other regions was implemented. Also, mass epidemiological investigations have been carried out for suspected cases, confirmed cases, clusters, and contacts to identify sources of spread to implement targeted control measures. Thus, these measures quickly started producing the desired outcomes. For example, the number of new confirmed cases per day declined from a high peak of 3,900 cases on 13 February 2020 to 323 cases on 27th February 2020 (9). Besides, at a COVID-19 news conference on February 24, 2020, the WHO leader, Bruce Aylward, said “China's made comprehensive non-pharmaceutical interventions, which have effectively prevented the transmission of the virus, providing important experiences for the global response to new virus” (10). Also, at 16:00 h on 28 February 2020, Dr. Wannian Liang, the China team leader and the expert team leader of the New Coronary Pneumonia Epidemic Response said: “the virus outbreak in Wuhan was curbed basic” (10). Moreover, Wilder-Smith et al. noticed that the unprecedented efforts by the Chinese government surpassed the previous efforts to combat sirs (11). Further, as of March 7, 2020, the total number of close contacts tracked was 6,740,388. Therefore, there is no doubt that the prevention and control measures implemented by China have been very effective and could serve as an example for other countries (10). Thus, it is against this background that this paper aims to summarize the foregoing intervention measures, from the perspective of social epidemiology, and try to explain how the COVID-19 outbreak in Wuhan, Hubei Province was effectively controlled. Also, this study aimed to explore the effectiveness of the ambitious non-pharmacology interventions taken by the Chinese government. It is therefore hoped that the findings of this study would provide references and suggestions for other countries in the progress of the epidemic.

## Methods

### Ethical Approval

In this study, it was not necessary to obtain the institutional review board's approval due to the use of publicly accessible data of published cases, and the project is not involved with patient consent.

### Data Collection

Public health intervention data were collected from January 11, 2020, to March 5, 2020. Thus, detailed intervention measures conducted by China in Hubei Province were described, along with the subsequent changes in trends and analysis of its effects. Data were extracted, tabulated, and analyzed in Microsoft Excel 2010 and drawn a graph by software of Graph Pad Prism (version 9.0). The following steps describe the data collection process for this study.

**Step 1:** Data were extracted from the information reporting system of the National Health Committee, issued by the health emergency office, between January 11, 2020 and March 5, 2020 (http://www.nhc.gov.cn/xcs/situationreportofCOVID-19). Specifically, extracted data included the cumulative number of confirmed COVID-19 cases (in the Mainland China, Hubei Province) and the number of daily new confirmed COVID-19 cases (in the Mainland China, Hubei Province, Wuhan City). In addition, the ratio of daily new confirmed COVID-19 cases to daily cumulative COVID-19 cases was considered as an indicator of the effectiveness of the interventions.

**Step 2:** Relevant important information and announcements were also collected from the official website of the Chinese government (https://www.gov.cn); National Health Commission of the People's Republic of China; announcements of the Joint Prevention and Control Mechanism for new coronary pneumonia of 2019 of the State Council on Implementing Measures to rescue Hubei Province; press conference on the progress of prevention, control and treatment of the epidemic situation of new coronary pneumonia of 2019 (http://www.nhc.gov.cn/xcs); the website of the People's Government of Hubei Province (http://www.hubei.gov.cn); the website of the People's Government of Wuhan (http://www.wuhan.gov.cn); Health commission of Hubei Province (http://www.wjw.hubei.gov.cn); Health Commission of Wuhan (http:www.wjw.wuhan.gov.cn); and Chinese Center for or Disease Control and Prevention website (https://www.chinacdc.cn/)and Hubei Provincial Center for Disease Control and Prevention website (http://www.hbcdc.com/). Additionally, relevant information was obtained from other media platforms such as news, articles, and reviews.

## Results

### Response Measures Summary and Data Analysis

A retrospective description and summary of the observed public health intervention strategies are presented in three stages as follows:

**Stage 1:** During the early stage of the outbreak, before 20 January 2020, the main strategy focused on preventing the exportation of COVID-19 cases from Wuhan and Hubei Province; and preventing the importation of cases by other provinces. The overall global was to control the source of infection as much as possible, block transmission, and prevent further spread to the other provinces. On Jan 26,30 provinces across the country initiated a first-level response to a nationwide level. Wuhan City organizes experts to analyze the condition, outcome of treatment, epidemiological investigation, preliminary laboratory testing, and efforts were made to identify the zoonotic source diseases (7). Information on the epidemic was sent to WHO on January 3, 2020, and the whole genome sequence of the SARS-CoV-2 virus was shared with World Health Organization and the United States and other countries on January 10, 2020 (12, 13). Subsequently, the first guidelines for COVID-19 diagnosis and treatment were formulated (14).

As we all know, since the outbreak of COVID-19, the Chinese government puts the people's life safety and physical health first, with firm courage and confidence, and adopting the most comprehensive, strictest, and thorough prevention and control measures to control the epidemic (15). For example, Wuhan public transport was closed and the National Health Commission of the People's Republic of China and other six departments issued the “Notice on Strictly Preventing Pneumonia Caused by the Transmission of New Coronavirus Through Transportation,” subsequently, all provinces across the country have successively initiated a provincial-level emergency response to major public health emergencies. Sixteen temporary treatment hospitals were opened by February 24, a large-scale health quarantine was implemented, social isolation at a whole society level was enforced, hygiene practices were encouraged, and every citizen was ordered to wear a face mask and keep a distance of 1.5 m from other individuals. These interventions were coordinated by an established Central Leadership Group for Epidemic Response and the Joint Prevention and Control Mechanism of the State Council. Furthermore, President Xi Jinping personally directed and deployed the prevention and work control, and requested that the prevention and control of the COVID-19 outbreak be the top priority of the government at the national level. In this regard, the prevention and control measures have been conducted quickly, from the early stages in Wuhan and other key areas of Hubei province. Table 1 shows the three phases in which these interventions were undertaken. Noteworthy, the novel coronavirus outbreak was included in the statutory report of Class B infectious diseases and management measures for Class A infectious diseases have been adopted on January 20, 2020, which marked the transition from the initial partial control approach to the comprehensive adoption of various control measures by the law (16).

**Table 1:** Details of public health intervention measures taken.

**Date (Stage I: from January 20 to February 15)**	**Measures**
2020-01-20	President of the People's Republic of China and have issued important instructions on the epidemic of a cluster of acute respiratory illness, saying that the safety and health of the people's should be put first, we will resolutely and firmly curb the spread of the epidemic and emphasize the need to disseminate timely information on the epidemic and deepen international cooperation.
2020-01-20	Coronavirus-disease-2019 was included in the statutory report of Class B infectious diseases on 20 January 2020 by the National Health Commission of people's public of China and timely release of information on the epidemic situation.
2020-01-22	The Central Committee of the Communist Party of China has urged Hubei province to immediately implement comprehensive and strict controls on the outflow of people of Hubei Province and Wuhan City.
2020-01-23 10:00 a.m.	Wuhan, according to the announcement of the epidemic prevention and control headquarters, the public transport was suspended, and the airport and railway station, and existing channel off Wuhan were temporarily closed.
2020-01-24	The state council held the conference on joint prevention and control mechanism of pneumonia epidemic caused by new coronavirus infection pneumonia spread situation.
2020-01-25 (The first day of the Chinese New Year)	President Xi Jinping personally hosted a conference of the standing committee of the political bureau of the Central Committee of the Communist Party of China, he put forward requirements on the prevention and control of the epidemic, made a comprehensive study of the situation, and promptly put forward the general demands of “strengthening confidence, working in the same boat, scientific prevention, and control, and taking targeted measures” as an overall goal. On January 25, members of the standing committee of the political bureau of the CPC held special meetings to launched the prevention and control of the epidemic, especially the treatment of patients.
2020-01-27	Prime Minister Li Keqiang entrusted by president Xi Jinping, and leader of the Central Leading Group for Response to the Epidemic, went to Wuhan to inspect and guide epidemic prevention and control work and expressed condolences on behalf of the CPC Central Committee and the State Council. On the same day, the Central Steering Group was stationed in Wuhan to comprehensively strengthen the guidance and supervision of the frontline epidemic prevention and control. Li Keqiang went to Jinyintan hospital, which was the largest number of confirmed and severe patients hospital, and made a video conference with medical staff in the negative pressure ICU, and communicated with doctors.
	Vice Prime Minister Sun Chunlan led the central groups to Hubei province to carry out the prevention and control of the COVID-19 guidance.
2020-02-02	Two improvised hospitals (“Huoshenshan, Leishenshan”) were beginning to build.
2020-02-03	Present Xi Jinping chaired a meeting of the Standing Committee of the Political Bureau of the CPC and pointed out the need to further improve and strengthen prevention and control, and believed an important speech.
2020-02-03	On Feb 3, medical rescue teams from 20 provinces were urgently selected to gradually take 13 exhibition centers in Wuhan transform them into “quadrangle hospitals” to make sure the mild patients were treated. Two days later, the first three quadrangle hospitals were opened and the first mild cases were admitted (there are 4,250 beds in the three hospitals).
2020-02-04	Wuhan temporary (“Huoshenshan”) hospital received the first batch of confirmed patients of COVID-19.
2020-02-05	Wuhan, completed three temporary hospitals and the national emergency medical rescue team to help Wuhan.
2020-02-06	Various measures to increase the supply of beds, through the deployment of external support forces, tapping local potential to increase the number of medical staff; by tapping the potential of existing designated hospitals, building new hospitals, and setting up centralized isolation points to increase the supply of beds, and by tapping the potential of local development and organizing 16 provincial (regional and municipal) counterpart support, to increase the relevant municipal and county medical forces. At the same time, we will continue to ensure the supply of key medical recuse resources (test kits, facemasks, and personal protective equipment) in accordance with the arrangements already made.
2020-02-06	The Central Steering Group headed by Vice Premier Sun Chunlan arrived in Wuhan, and launched a comprehensive “war on the front-line prevention and control of the epidemic”.
2020-02-07	Wuhan, Hubei province: the temporary hospital will carry out acceptance inspection and complete construction (more than 10 days).
2020-02-09	Wuhan: another (“Leishenshan”) temporary hospital was receiving patients.
2020-02-10	President Xi Jinping conducted guidance on the situation of the epidemic in Beijing, and connected video conferences with the medical staff of Wuhan Jinyintan hospital, union medical hospital, two temporary hospitals.
2020-02-12	Nearly 20,000 medical workers were been sent to assist Wuhan, Hubei province, and other areas for medical treatment. We will organize 19 provinces to provide more support to medical workers in other areas of Hubei province while doing local prevention and control work. Meanwhile, the outbreak site of the Hubei epidemic bureau should take the same measures immediately as Wuhan.
2020-02-12	The national health commission held a meeting of leading party members to study and put forward “precise prevention and control measures” for COVID-19. Hubei province, especially Wuhan, is still the top priority in epidemic prevention and control. We should focus on solving the problem of insufficient beds and medical personnel, increase the supply of critical care beds, ensure smooth transfer channels for treatment and treatment, strictly implement the measures of centralized management of “four types of person,” and ensure the suspected infection cases were cleared.
2020-02-14	Vice prime Sun Chunlan, who has been working on the frontlines in Wuhan, said: we will make further arrangements to defend Wuhan and Hubei. Based on the goals “Save the patients and prevent the disease were spread,” We will strive to make sure that all of the confirmed patients were received, all of the suspected patients received nucleic acid testing, all of the febrile patients receive were tested, all of the close contacts are isolated, and all of the community villages implement closed management with 24 h. We must ensure that all receivables patients are collected, earnestly implement the “four early” measures, and resolutely control the source and cut off the way of transmission. We will continue to integrate traditional Chinese and western medicine, move forward, care for medical workers, and increase treatment for minor and severe cases.
2020-02-15	This time, for the epidemic prevention and control work in Wuhan and Hubei province, it is the most critical stage. In the most critical phase, Wuhan in Hubei province is still the main battlefield. At present, it has opened nine hospitals with more than 6,960 beds and 5,606 patients in the hospital. Excellent medical workers were sent to Hubei and Wuhan from all places. (1) In terms of reducing the infection rate, Hubei province, especially Wuhan city, has formulated and implemented local prevention and control measures according to the actual situation, strictly implemented the “four early” measures, and effectively completed the classified centralized management of “four types of person.” (2) To push the prevention and control forces to sink into the community, precise management, make use full of the power at the community level, to curb spread from communities. (3) In terms of improving the admission rate, distinguish different situations, classify and treat all the existing patients with confirmed severe diseases, including those with clinical diagnosis, to be treated in a designated hospital, and those with confirmed mild diseases can be treated and observed at the isolation point.
2020-2-16 Stage II: From February 16 to 19	On 19 February P.M, the Hubei province people's government website issued three most strictly notices within 1.5 h. That is, urban and rural communities and village groups were adopted closure management measures: residential areas are under the strictest closed management 24 h; Lanched mass health screening for all residents in Wuhan (only 3 days, February 16–19): Prevent the widespread of the coronavirus in Communities, the empower communities to curb the spread of the coronavirus base on the following principle: conclude the exactly cases number, leave no one unscreened constantly, each day to ensure full coverage, no leave blind spots (1) Strengthening the epidemiological investigation of the “four categories of people” (exposed infection persons, close contact of the diagnosed person, suspected cases, and confirmed cases) (2) Strengthening the management of key areas. All non-essential public places shall be closed and all mass gathering activities should be stopped (3) Strengthening comprehensive health screening of residents: The community (village), community, residential area, building, workplace, and other grass-roots units with confirmed (including clinical diagnosis) cases of COVID-19 should be firmly isolated in a closed and hard manner for 14 days Carry out dynamic rolling screening for all residents to ensure that “no one household was missed, no one left behind, and no one left behind.” Ensure all communities were full coverage Strengthening the closure management of rural village groups requires that the natural village group (village bay) should be used as the unit to implement hard isolation. Each household may send one person every 3 days to purchase necessary daily necessities (4) Strengthening the management of key areas. All non-essential public places shall be closed and all mass gathering activities shall be stopped.
Stage III: From February 23 to March 5	Focusing on reducing the accumulation of cases, completely controlling the outbreak, and eliminating new confirmed cases, the China government begin to strike a balance between containing an epidemic, sustainable economic, and social development. China has taken strict measures to delay the start of school, return to work flexibly, travel on different dates, and monitor health and personnel management. Wuhan and Hubei provinces, continue to strictly control the traffic transitions, such as controlling the entrances and exits, and other provinces in China to prevent the risk of imported cases.
2020-2-25	Starting from February 25, entry-exit health and quarantine work have been comprehensively strengthened, with strict health inspections, temperature monitoring, medical inspections, epidemiological investigations, medical screening, and sampling monitoring for entry-exit personnel to prevent the cross-border spread of the epidemic.
2020-3-5	China's efforts to contain the spread of the epidemic are being accompanied by a gradual resumption of productive activities in the economy, education, and all sectors of society. In Wuhan and other key areas of Hubei Province, treatment and interruption should be highlighted, and the strategy of continuing to do solid work and doing fine should be emphasized. Based on the first-stage strategy, that is “all tests should be performed, all receivables should be collected,” and “all treatments should be treated.” The China government will apply a targeted region, to continue to strengthen epidemiological investigation, case management, and prevention and control of clusters of COVID-19 in high-risk areas.

Thus, on January 22, 2020, the State Council, and the Communist Party of China (CPC) central committee “made an official announcement” that Wuhan City, a metropolis of 11 million people, of Hubei Province, must shut down traffic transportation to control the flow of people. Further, on January 23, 2020, the authorities in Wuhan announced the lockdown of the City, which implied shutting down all public transportation, canceling flights and trains, and closing schools and factories (17, 18).

In addition, on January 27, 2020, Premier Li Keqiang, member of the Standing Committee of the Political Bureau of the CPC Central Committee, Premier of the State Council, and leader of the Central Leading Group for Response to the Epidemic, went to Wuhan to inspect and guide epidemic prevention and control work and expressed condolences on behalf of the CPC Central Committee and the State Council. Moreover, Li Keqiang went to Jingyintan Hospital, which had the largest number of confirmed and severe cases, and made a video conference with medical staff in the negative pressure ICU, and communicated with frontline health workers. Similarly, the vice prime minister, Sun Chunlan, led the central groups to Hubei to carry out the prevention and control measures of the COVID-19. Thus, by January 29, 2020, 31 provinces and autonomous regions and municipalities across China had launched the first- level of emergency response against the COVID-19 epidemic.

On 3 February 2020, medical rescue teams from 20 provinces were urgently selected to gradually transform 13 exhibition centers in Wuhan into “temporary treatment centers” to ensure that all patients with mild symptoms were treated. Two days later, the first Fangcang shelter hospital was opened, and the first cases with mild symptoms were admitted (there were 4,250 beds in the three hospitals). In addition, on 4 February 2020, “Huoshengshan” improvised hospitals in Wuhan began to accept the patients. On 7 February 2020, another temporary hospital underwent an acceptance inspection and was completed (10 days) in Wuhan. Later, on 10 February 2020, President Xi Jinping conducted a video conference in Beijing with the medical staff of Wuhan Jinyintan Hospital and Wuhan Union Hospital, and “Huoshenshan Hospital” in Wuhan to give guidance on the prevention and control of the epidemic (19). On 12 February 2020, nearly 20,000 medical staff were sent to assist in providing medical services in Wuhan, Hubei Province, and other areas (20). Therefore, the Chinese government organized medical staff from 19 provinces to take the form of “one province support to one region” to provide support to other areas of Hubei Province (It is a national strategy in China for a province or major city to assist in a designated region that needs help). These medical staff carried with them ventilators, electrocardiograph monitors, and ECMO (Extracorporeal Membrane Oxygenation) other medical and protective equipment.

**Stage 2:** On 16 February 2020, epidemic prevention and control measures in Wuhan and Hubei came to the most critical stage. The main strategy was to reduce the intensity of the outbreak and slow down the occurrence of new cases. Thus, in Wuhan and other key areas of Hubei Province, the focus was on treating patients aggressively, reducing the infection rate and deaths, and preventing cases from exporting the infection to other places. Nevertheless, in other provinces, the focus was on the prevention of importation, containment of disease transmission, and implementation of joint prevention and control measures. On 15 February, at present, the epidemic prevention and control work in Hubei and Wuhan has reached the most urgent time, Wuhan was still the main battle area. As of on 24 February 2020, nine new hospitals with more than 6,960 beds were opened in Wuhan, and 5,606 patients were accomodated. Accordingly, more medical workers from all places in China were sent to these hospitals to offer medical rescue services.

The state council information office of the Public of China press conference was held in Wuhan, Hubei to introduce the epidemic prevention and control and medical treatment work organized and carried out in Hubei.

As regards reducing the infection rate, Hubei Province, especially Wuhan, formulated and implemented local prevention and control measures according to the actual situation. Therefore, “four early stages” measures were strictly implemented (early detection, reporting, quarantine, and treatment). Also, and effectively completed the classified centralized management of “four categories of vulnerable people” (confirmed cases, suspected cases, febrile patients who might be carriers, and close contacts) was effectively completed classified centralized management. The prevention and control measures were conducted in the community, with precise management, and it was imperative to make full use of the power at the community level to curb the spread from communities.

Also, medical conditions and resources have been greatly improved, so the admission rate was improved greatly, and COVID-19 cases were classified as mild or severe; hence, management of patients was performed according to these categories of patients in designated hospitals (21). In addition, a nucleic acid test was conducted on suspected cases and the results were released the same day. Thus, when the test results were positive, patients were transported to designated hospitals depending on the severity of the symptoms. Noteworthy, all patients, those with mild or severe symptoms, and the asymptomatic patients were admitted. At least one designated hospital was strategically placed in each district/country.

Most importantly, the strategy in Wuhan and other critical areas of the epidemic was to “leave no one unscreened.” Therefore, closed community management every 24 h was launched in Wuhan while conducting a massive medical screening of COVID-19 infections (22). On the afternoon of 16 February 2020, the people's government of Hubei Province issued the three strictest announcements within 1.5 h. Therefore, the following actions were carried out according to the preceding strictest orders.

(1) Urban and rural communities and village groups adopted closure management measures. Thus, residential areas were under the strictest 24 h of closed management. Also, dragnet dynamic rolling screening was performed for all residents (only 3 days, 16–19 February 2020). To find the exact number of cases based on the foregoing strategies, everyone was screened; and also the epidemiological investigation of the following “four categories of vulnerable people” was strengthened; (2) Strengthening the management of key areas. This demanded closing all non-essential public places and banning all mass gathering activities; (3) Strengthening comprehensive health screening of residents: (a) the communities (villages), residential areas, buildings, workplaces, and other grass-root units with confirmed (including clinical diagnosis) cases of COVID-19 were firmly isolated in a closed and hard manner for 14 days. (b) The dynamic rolling screening was carried out for all residents to ensure that no one was left unscreened. Therefore, it was important to ensure that all communities and residents accepted medical screening. (4) Closure management of rural village groups was strengthened and this required that the natural village groups (villages) be used as the units for implementing hard isolation. Each household could send one person every 3 days to provide daily necessities to the family members who were in isolation.

**Stage 3:** In this third stage, the rapid rise of the epidemic situation in Hubei Province and Wuhan City has been contained. The situation of the epidemic situation in the country except Hubei Province is generally stable. In mid of March, the daily new cases were controlled within single digits, and the epidemic prevention and control achieved an important process. In accordance with the development of the epidemic prevention and control situation, the Central Committee of the Communist Party of China has made a major decision to coordinate epidemic prevention and control, economic and social development, and orderly resume work and production. Wuhan and Hubei Province continued to strictly control traffic transitions, such as controlling entrances and exits. Similarly, other provinces in China are also trying to prevent the risk of imported cases from other provinces and countries. These detailed measures are summarized in Table 1.

### New Confirmed Cases Situation

**Figure 1:**
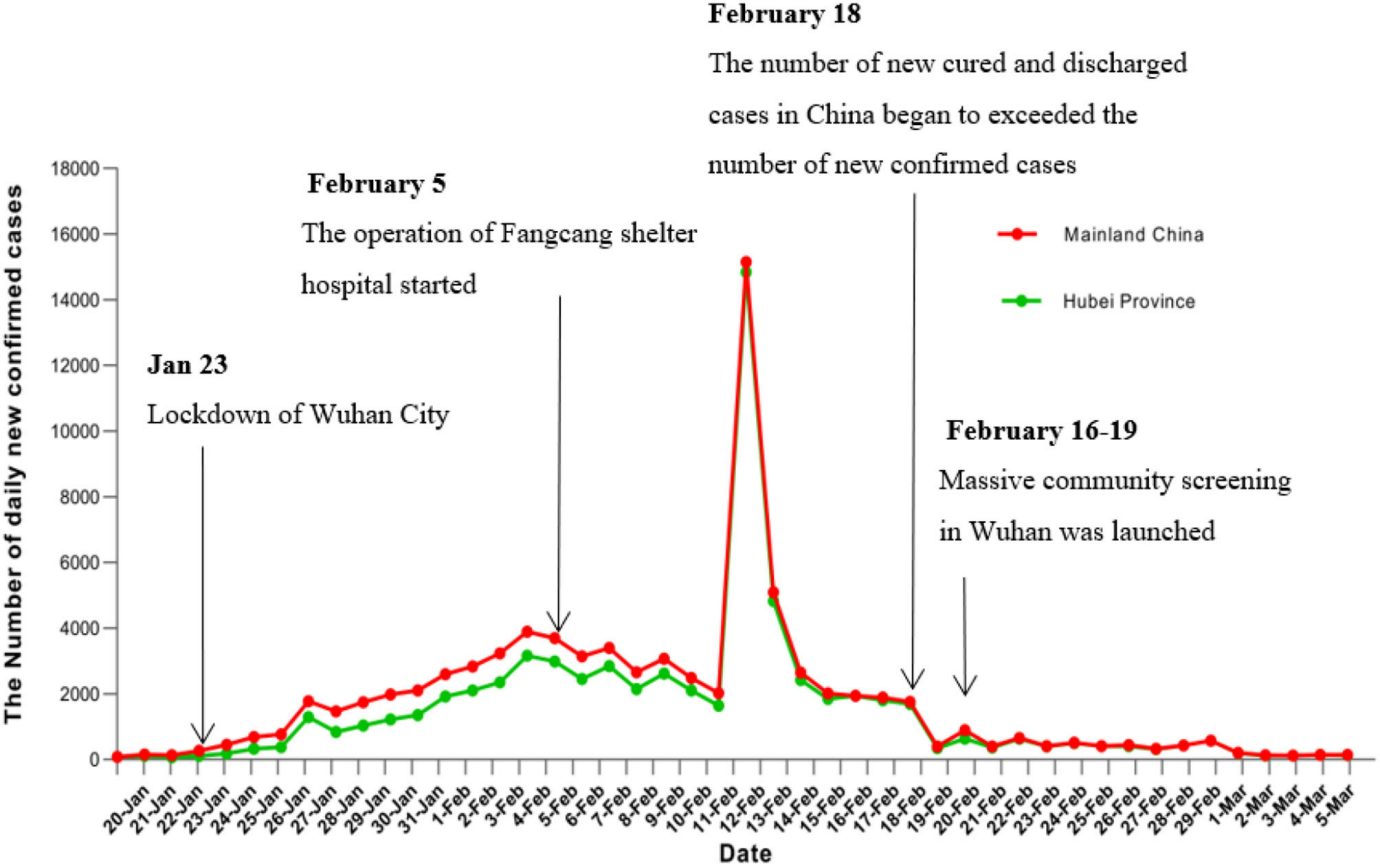
Situation daily confirmed cases number of COVID-19 in China mainland and Hubei province (from January 20 to March 5).

As shown in Figure 1, on 12 February, the number of confirmed cases was taken from the highest day, and the new confirmed cases number rose sharply before 20 January 2020, and this trend almost continued to the third week, but the epidemiological curve shows that the extraordinary intervention measures implemented by China government directly led to flat declines or lower levels of transmission from 17 to 20 February 2020 in Hubei province. Additionally, the epidemiological curve declined sharply on 19 February, and this positive outcome is attributed to the lockdown of Wuhan City, and the opening of new hospitals in Wuhan, with more than 6,960 beds, which accommodated 5,606 patients. Accordingly, more medical workers from all places in China were sent to these hospitals to offer medical services. Therefore, the hospital admission capacity was raised greatly (23). Besides, during the stage II period, the medical conditions and rescue resources were substantially improved; the centralized isolation and treatment of “four categories of vulnerable people” was implemented, and the control measures of the community using 24-h close management were constantly upgraded. Thus, the risk of infection was greatly reduced. Noteworthy, to promote the implementation of the five “100%” work goals, resolutely curb the spread of the epidemic, the Wuhan City government launched a mass screening in four thousand communities with 4.21 million residents between 16 February and 19 February 2020. A door-to-door and individual-to-individual universal symptom survey to single out suspected cases in the community, which was strongly associated with further reductions in the spread of COVID-19 in Wuhan. Besides, to further reduce the risk of infection in the community, Wuhan city adopted the methods of a closed community, closed residential areas, all residents were asked home isolation, the government is responsible for centralized distribution of living materials and other means. Therefore, the epidemiological curve shown in Figure 1 proves that the preceding prevention and control measures are successful and directly lead to desired outcomes.

### Change Trends of New Confirmed Cases

**Figure 2:**
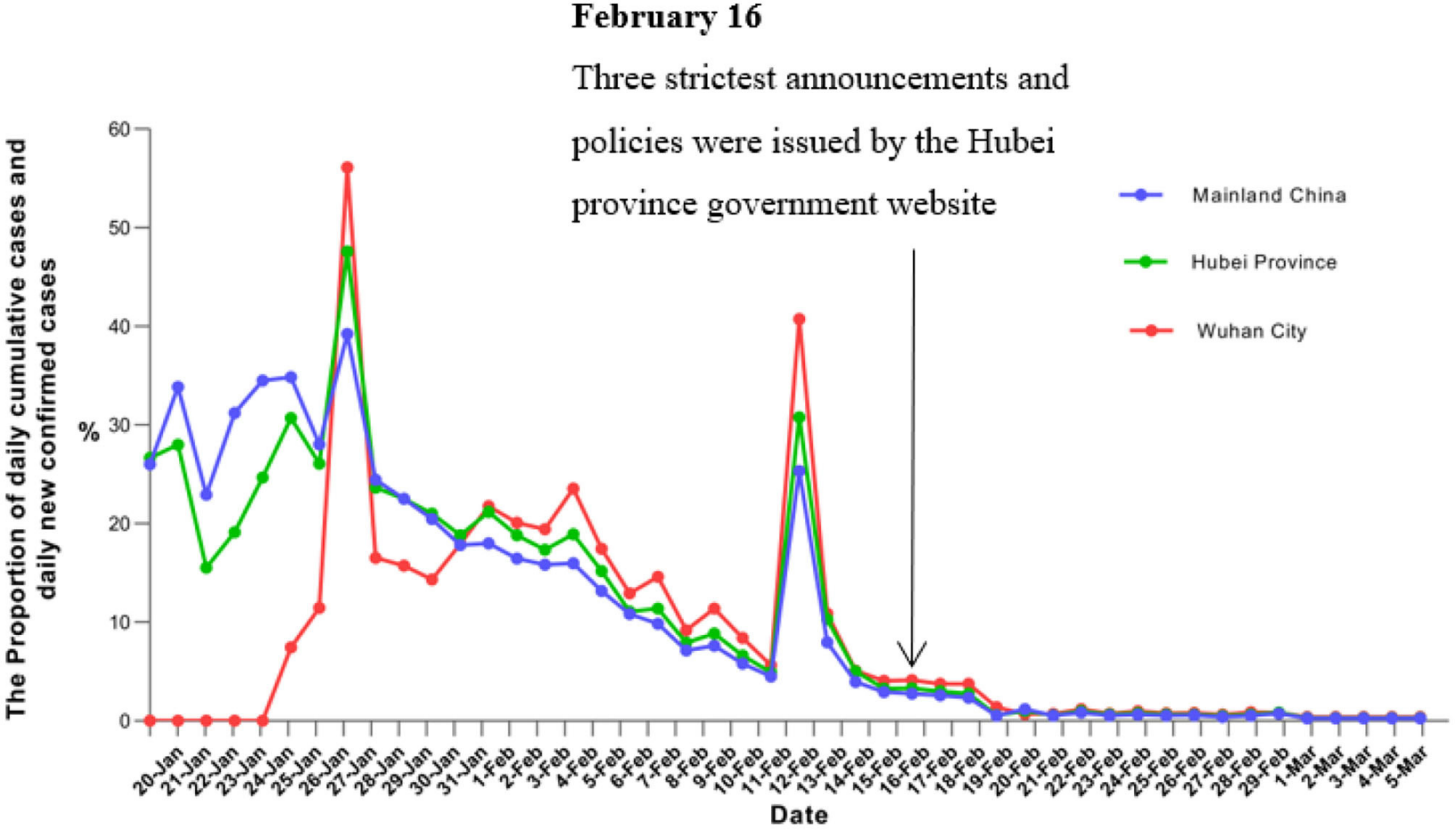
Change trends of daily newly confirmed cases and daily cumulative cases.

Figure 2 also presents a trend that peaks near a plateau and then declines on 20 February 2020. This changing trend also indicates that the three strictest announcements and policies achieved a smooth decline in the epidemic. Additional measures included restrictions on inter-city travel during the stage I period, early identification, and isolation of cases as well as restrictions on personal contact and measures of social alienation. In addition, the expansion of services for PCR testing improve day by day, continue to improve nucleic acid detection capabilities, and enhance the capacity of test kit supply, many commercial organizations have also joined nucleic acid testing and the capacity of nucleic acid testing to 2, 5 000 tests per day on 26 February 2020, in Wuhan (24). It added that there was inventory enough to fulfill testing for about two million people.

So greatly shorten the diagnosis time of patients and reduce the risk of transmission. Moreover, community villages implemented 24-h closed management, each resident who was isolated in the home, the government centralized distribution of living necessities supply to them, etc. Twenty-four closed community management and control measures continue to keep in Wuhan. These comprehensive intervention measures contributed to the decline in the epidemiological curve shown in Figure 2.

## Discussion

COVID-19 outbreak is the world's most serious infectious disease epidemic since the 1918 pandemic and the most challenging global public health emergency since the end of the Second World War. Since the founding of the People's Republic of China in 1949, the COVID-19 epidemic is a major public health emergency with the fastest spread, the widest range of infections, and the most difficult prevention and control has occurred (25). Thus, it has been the biggest battle to prevent and control its rapid spread across the country, which required the involvement of all the people in China. This study, therefore, systematically collected and analyzed data to evaluate the prevention and control measures implemented by China. The purpose was to inform the reader how China government has thus far performed in the prevention and control of COVID-19 and to highlight non-pharmacology measures that could effectively, timely reduce the spread of the COVID-19 pandemic globally. Thus, this is consistent with the WHO declaration in the early stages of the outbreak that COVID-19 is the Public Health Emergency of International Concern, based on the International Health Regulation (2005), as he said that China has taken extraordinary and powerful measures, and China has provided an example for responding to the epidemic for many aspects, as it is an extraordinary event to constitute a public health risk to the states through the international spread of the disease, and this potentially required coordinated international cooperation and response to jointly control the virus (26, 27).

Therefore, the international community needs to work together in the prevention and against the COVID-19 pandemic (28). Nevertheless, some countries, such as Sweden and The United Kingdom, still take a vague attitude about this outbreak, such as “herd immunity” (29). Also, some people may refuse to wear face masks in other countries. Mankind will eventually defeat the epidemic, but a major public health emergency will not be the last time for mankind (30, 31).

Experience has shown that non-pharmacology interventions can successfully reduce or even stop transmission in some cases. Even in the face of widespread transmission, China's experiences have shown that if we keep calm, take action immediately, and implement a systematic identification of cases as well as contact tracing, then we could change the course of the pandemic, prevent people from getting sick, and prevent the most vulnerable from dying.

To the best of our knowledge, the main four early strategies: “early identification, early report, early isolation, and early treatment” were critical to the containment of the COVID-19 epidemic in China. These efforts were accomplished via a process of “lockdown Wuhan” to conduct two rounds of community-based mass screening of its 4.21 million households, finally, the infection rate was minimized greatly in Wuhan and achieved success based on the principle of “leave no one unscreened household unchecked” and to ensure that there are no new potential sources of infection occurred (32).

In addition, very strict containment measures for the COVID-19 outbreak included the “four categories of vulnerable people” being accepted screen nationwide, and the community-based epidemic prevention and control were used as the basic unit to carry out a carpet-style investigation by combining on-site investigation with self-report. Fully implement body temperature screening and health quarantine in various places, including travel venues (airports, train stations), strengthening the monitoring of fever cases in medical institutions and direct online reporting of infectious diseases, implemented 2 h direct online reporting, 12 h feedback of PCR test results, complete on-site epidemiological investigations within 24 h, and timely discover and report confirmed cases and universal symptom survey (33).

These active actions were implemented successfully due to strong coordinated efforts by the Chinese government in cooperation with residents. To date, the epidemiological data in this study has shown that China effectively reversed the situation of the epidemic, and China took more than a month to initially contain the spread of the epidemic, and thousands of people were protected from the virus and also save valuable time to the world (32, 34).

Yang et al. (35) used the population migration data on 23 January 2020, and the latest COVID-19 epidemiological data to integrate the classical infectious disease prediction model to predict the epidemic trend, which showed that the response measures are taken by the Chinese government effectively avoid the spread of the epidemic. If these public intervention measures had been delayed for another 5 days, the epidemic trend would have tripled, and the spread of the disease to many places could have grown exponentially in one region, hence devastating for humans (36). Recently, some studies have examined the effects of the shutdown of Wuhan public transportation (37, 38), strong travel restrictions, and prohibits mass gatherings (38), massive health quarantine conducted in the airport (39), isolation of cases for 14 days, and contact tracing on the containment of the disease by 5G network big data technology platform tracking individual health codes (40). Thus, Lai et al. developed a modeling framework that estimated that without non-pharmaceutical interventions, the number of cases would have been 67-fold higher by 29.02.2020 (41). In April, Pan et al. use the real case data of Wuhan to systematically and completely analyze the impact of the continuous improvement of public health intervention measures on the epidemic trend in Wuhan. These studies revealed that public health interventions play an important role in the prevention and control of new emerging infectious diseases. Accordingly, in China, a combination of the preceding non-pharmaceutical interventions and traditional pharmaceutical interventions China achieved the strongest and most rapid effect (32).

There are two positions in the fight against the epidemic, one in the hospital to rescue patients, and the other in the community prevention and control position. The community played an important role in the prevention and control of the epidemic In, In this fight, medical resources from all over China have been mobilized to support the medical treatment of patients in Wuhan and Hubei Province. From New Year's Eve on 24 January to 8 March, a total of 346 national medical teams, 42,600 medical staff, and 965 public health workers from 29 provinces, and Xinjiang production and construction corps were used to assist Wuhan and Hubei Province (42). Besides, it took <10 days to construct two temporary hospitals (43, 44).

As of 28 February 2020, 16 new shelter hospitals were opened to centralize the treatment of patients based on the principle that all patients could be treated. Therefore, the outbreak in China, including the hardest-hit Hubei Province, has been well under control with high-level measures for prevention and control, and temporary hospitals playing the role of isolating non-severe patients. Specifically, the shelter hospitals in this epidemic have created a new model for China to respond to public health emergencies and crises, and to rapidly expand medical resources. That is, as Wang Chen, who is an academician with the Chinese Academy of Engineering said “The shelter hospitals” admitted only patients with mild symptoms, which apart from quarantining the patients, also created room for regular hospitals to treat several types of patients, provided high-quality medical treatment and care, and fulfilled an important triage function (45–48).

In response to the prominent contradiction between the rapid increase in the number of patients in the early stage of the epidemic and insufficient bed resources, the Chinese government did everything possible to reserve beds, and concentrate resources and efforts to build and expand designated critically patients hospitals in Wuhan, and to ensure that all cases could be treated on time. Wuhan and Hubei Provinces were treated as a priority for medical materials needs, and many provinces (a total of 19 provinces) were organized to help one region (49). In addition, make full use of new technologies such as big data and artificial intelligence to enhance contact tracing, focus group management, resource allocation, identification of the source of suspected cases, and conducting epidemiological investigations in the community across the country. The data and information system for epidemic prevention and control, based on the people's public security big data system, were an important “weapon” for Wuhan to win over the battle of epidemic prevention and control.

Additionally, the Wuhan government proposed a large step to ensure that 100% of confirmed patients were received and treated at the hospitals, 100% of suspected cases were undergoing nucleic acid-based testing, 100% of patients with fever were detected, 100% of close contacts persons were isolated, and community villages implemented 100% 24 h closed management (50).

This study, therefore, suggests that non-pharmacological public health interventions are crucial to stopping the global spread and progression of COVID-19 in the absence of targeted vaccines and specific drugs. Also, a series of improved public health intervention measures in Wuhan has been identified by some studies (33, 51–54). These findings may help inform the public health policies in other countries and regions as regards response to the global COVID-19 pandemic. However, the disadvantages of containing COVID-19 in China must be discussed in this study. For example, social isolation between people, and maintenance of strict intervention measures lasting more than 2 months in Wuhan, particularly physical distancing, and thus brings huge socioeconomic costs (55).

First, since the COVID-19 outbreak, the government of China implemented the right to good health a priority. China always adheres to the concept of a community of human destiny, and urges the international community and country leaders to join in the fight against stigma and the politicization of public health issues, but minimize the impact of the epidemic on the economic burden and society, hence protecting the rights of vulnerable populations (56). Second, any possible violation of human rights while carrying out social isolating measures to prevent and control the spread of the pandemic should be discussed. Thus, as a matter of fact, China's mode of response is the most comprehensive, stringent, and thorough regardless of exposing herself to a period of economic decline or even a short-term “shutdown.” This is consistent with an editorial comment in the Lancet, “China's success has come at a huge social and economic cost, and China must make tough decisions to strike the best balance between health and economic protection of its citizens (56).” Besides, isolation and travel bans are often the first response against new infectious diseases; hence, the Chinese authorities also ordered a 14-days quarantine mandatory for any person who has recently visited the Hubei province. However, in the United States of America (USA), quarantines and ban massive gatherings, like these traditional tools are usually of limited utility for highly transmissible diseases, and if it imposed with too heavy a hand, or in a hazardous manner, they can be counterproductive (57). Therefore, the implementation of these strategies in other countries or regions during the current pandemic needs to carefully consider other contextualization principles such as ethics, cultural, and socioeconomic factors.

## Lessons and Future Gaps

Heavy lessons have been learned from the COVID-19 outbreak. In addition, this outbreak has sounded an alarm, revealing that China has an obvious knowledge gap in the public health government system and the mechanism of public health emergency management. We summarize the following lessons based on a very limited understanding.

First, very important questions exist: Who should report an epidemic outbreak? Where to report? And who should make these important decisions? To our knowledge, the Centers for Disease Control (CDC) in China has been established as the most effective reporting system since the SARS outbreak in 2003. This has been the case because the China government gives enough importance to the disease control and public health system, which was the best reporting system in the world at that time. For instance, the beginning of a number of high-level biosafety laboratories began, and high-end equipment continued to be deployed (58). Also, laboratory testing capacity, epidemiological investigation capacity, and on-site disposal capacity have been rapidly improved. Unfortunately, it seems that these did not develop fully during this SARS-CoV-2 pandemic (58).

We believe that, when people are facing a sudden outbreak of an unknown infectious disease, it is better to quickly take note and inform the public in a manner that the public health and emergency response could be implemented at an opportune time. According to the situation in early January 2020, the data integration of multiple levels may not have been realized in Wuhan. For example, the data of the infectious disease report system of the local CDC, the diagnosis and treatment data of the medical and administrative system, and the data of various research teams were not integrated on time in the early outbreak of Wuhan (59).

Second, in the early stages of the outbreak, the CDC did not develop good quality production of detection reagent in a very short time and did not ensure good production quality. In this sense, the case sample collection became very difficult. Thus, each communication and consultation channel between the local CDC and medical institutions did not have good collaboration in the early stages, which badly affected the early screening and diagnosis of the infected people (60).

Finally, in the very early stages, due to panic and anxiety among people, and lack of proper screening methods, residents with fever, influenza, and COVID-19 infection cases were admitted into fever clinics, resulting in a large number of patients with COVID-19 becoming the transmission source within the clinic setting, and cross-infection at the community level, hence putting the communities at a huge risk situation of was transmission. Obviously, the roles of general practitioners (GPs) and the community-level health service system may not have been fully developed in this crisis outbreak.

## Strengths

- In the context of the global public health crisis, this is the first study to describe China's practices and lessons in fighting the epidemic of COVID-19. It could provide valuable insights for pandemic control and public health emergency management in other countries and regions.
- This analysis shows that NPIs (nong-pharmaceutical intervention) measures initiated the Spring Festival holiday including the unprecedented Wuhan city Lockdown decisions and launched the level −1 emergency response at a national level were strongly associated with a reduction in case numbers during the COVID-19 epidemic in China.
- This study provides a new research perspective on public health emergency management in China and contributes to the relevant research in this field.

## Limitations

In this study, several limitations must be acknowledged. First, information on cured cases and death cases or unascertained cases (asymptomatic cases or patients with mild symptoms who could recover without seeking medical care) was not collected, so this was not discussed in the measures regarding medical treatment situations. In particular, the clinical features of the cases so that this shortcoming may not conducive to guide the control of the current Italian epidemic.

Second, we could not examine the impact of other factors of the national emergency response because other provinces were introduced simultaneously initiated across China. Because suspending intra-city public transport, and banning social public gatherings, home quarantine which was introduced at different times in different places, were also associated with overall containment virus transmission.

Finally, mental health sciences including psychiatry reach can play a key role in comforting individuals and front-line medical workers during the COVID-19 epidemic, and their family members. However, although Wuhan City had been shutting down transportation for more than 2 months, we did not discuss mental health services for patients, front-line medical workers, and their families. Therefore, future research should assess these impacts of COVID-19 prevention and control on the mental health issue.

## Main Findings of the Study

**(1) Prevention and control strategy**
  - First stage: (27 December 2019 to 19 January 2020) China Government respond promptly to emergencies 27 December 2019 to 19 January 2020, China reported the epidemic as soon as possible and took prompt action to carry out etiological and epidemiological investigations to stop the spread of the epidemic. Proactively notify the World Health Organization, the United States, and other countries of the epidemic situation in a timely manner, and publish the genome sequence of the new coronavirus to the world.
  - Second stage: (January 20 to February 20) General Secretary of the CPC Central Committee, President of the People's Republic of China, and Chairman of the Central Military Commission Xi Jinping made important instructions on the pneumonia epidemic caused by the new coronavirus infection, pointing out that people's safety and health should be put first, and the spread of the epidemic should be resolutely curbed. Wuhan has deployed four categories of vulnerable people for centralized management, carried out door-to-door screening of all residents, and conducted centralized isolation and virus testing for those with suspected symptoms. Wuhan City completed and opened the first batch of 3 Fang hospitals. During this period, the daily diagnostic testing capacity has further increased to about 20,000 cases. Big data; Artificial intelligence technologies were applied to identify confirmed cases and their close contacts and to monitor the body temperature of people flow. On 18 February, the number of newly cured and discharged cases nationwide exceeded the number of newly confirmed cases, and the number of confirmed cases began to decline.
  - Three stage: (21 February to 5 March) The rapid rise of the epidemic situation in Hubei Province and Wuhan City has been contained. The epidemic situation in the whole country except Hubei Province is generally stable. In March, the daily new cases were controlled within single digits, and the epidemic prevention and control achieved important results in stages. According to the development of the epidemic prevention and control situation, the Central Committee of the Communist Party of China has made a major decision to coordinate epidemic prevention and control, economic and social development, and orderly resumption of work and production.
**(2) Lessons**
  - At the very early in the virus epidemic, the reporting of major national public health epidemics to the public was not timely and appropriate, and the initial scientific prevention and control plan was lacking. After the outbreak of the epidemic, the emergency response mechanism is difficult to deal with major public health emergencies that threaten people's health. The problem of insufficient nucleic acid detection capacity in the early stage of the epidemic.
  - Communication and consultation channels between the local CDC and medical institutions did not have good collaboration in the early stages, which badly affected the early screening and diagnosis of virus-infected patients and thus the cases were been delayed diagnosis.
  - After the epidemic outbreak, there is no implemented hierarchical diagnosis and treatment in Wuhan, and the functions of the community health service system have not been effectively developed, thus, forming a crowded hospital and greatly increasing the risk your cross-infection.

## Conclusions

The NPIs taken by China play a decisive role to control the spread of novel coronavirus outbreaks. Further research and action are needed to ensure a sufficiently sensitive surveillance system and strong response mechanism, including the establishment of a highly accessible laboratory network, maintenance of awareness of both primary healthcare providers and the public, and regular training and exercise of local Centers for Disease Control and Prevention and general practitioners in the community-level.

## Data Availability Statement

Publicly available datasets were analyzed in this study. These can be found at: http://www.nhc.gov.cn/xcs/yqtb/list_gzbd_3.shtml.

## Author Contributions

SC and HX: conceived, designed the analysis, and conceputalizaion. SC and YZ: performed the analysis, wrote the paper, methodology, and writing—original draft. YZ and XZ: data curation. SC: formal analysis. HX and XZ: supervision. SC, AK, and HX: writing—review and editing. All authors contributed to the article and approved the submitted version.

## Funding

This study was supported by the School Scientific Foundation of Guizhou Minzu University [Grant No. GZMU ([2019]YB05)].

## Conflict of Interest

The authors have declared that no competing interests exist.

## Publisher's Note

All claims expressed in this article are solely those of the authors and do not necessarily represent those of their affiliated organizations, or those of the publisher, the editors and the reviewers. Any product that may be evaluated in this article, or claim that may be made by its manufacturer, is not guaranteed or endorsed by the publisher.
